# Characterisation of the complete chloroplast genome of *Gentiana delavayi* Franch. (gentianaceae), a medicinal plant in southwest of China

**DOI:** 10.1080/23802359.2019.1677196

**Published:** 2019-10-18

**Authors:** Ai-En Tao, Fei-Ya Zhao, Cong-Long Xia

**Affiliations:** a College of Pharmacy and Chemistry, Dali University, Dali, China;; b School of Medicine, Tourism and Culture College of Yunnan University, Lijiang, China;; c Key Laboratory of Yunnan Provincial Higher Education Institutions for Development of Yunnan Daodi Medicinal Materials Resources, Yun, China

**Keywords:** *Gentiana delavayi*, chloroplast, Illumina sequencing, phylogeny

## Abstract

*Gentiana delavayi* is a medicinal plant commonly used in southwest of China. In this study, we sequenced the complete chloroplast (cp) genome sequence of *G. delavayi* to investigate its phylogenetic relationship in the family Gentianaceae. The chloroplast genome of *G. delavayi* was 151,569 bp in length with 37.8% overall GC content, including a large single copy (LSC) region of 81,177 bp, a small single copy (SSC) region of 16,921 bp and a pair of inverted repeats (IRs) of 25,471 bp. The cp genome contained 114 genes, including 78 protein coding genes, 30 tRNA genes, and 4 rRNA genes. The phylogenetic analysis indicated *G. delavayi* was closely related to *G. stipitata*.

*Gentiana* L. is a great genus of the Gentianaceae family, which includes 362 species and has been classified into 15 sections in the world. Most of them are widely distributed throughout the northern hemisphere (Ho and Liu 1990; Ho and Pringle 1995). There are 248 species in China (Ho et al. 1995). Plants of this genus have been widely used in traditional Chinese medicine for thousands of years (Jiangsu New Medical College 1977). Among these species, *G. delavayi* is widely distributed in southwest China which have been used in local medicine (namely ‘Bai Yao’) for treatment of inflammation, hepatitis, rheumatism, and cholecystitis (Wang et al. 2009). However, up to now for such medicinal plant, many studies have mainly focussed on describing its chemical compositions (Wang et al. 2009; Zhang et al. 2019) and pharmacognostic studies (Yang et al. 2017), with little involvement in its molecular biology, so that no comprehensive genomic resource is conducted for it. Here, we report the chloroplast genome sequence of *G. delavayi* and find its internal relationships within the family Gentianaceae.

Fresh and clean leave materials of *G. delavayi* were collected from Dali county, Yunnan, China (N25.61°, E100.49°), and the plant materials and a voucher specimen (No. TAE02) were Tourism and Culture College of Yunnan University (Lijiang). Total genomic DNA was extracted using the improved CTAB method (Doyle 1987; Yang et al. 2014), and sequenced with Illumina Hiseq 2500 (Novogene, Tianjin, China) platform with pair-end (2 × 300 bp) library. About 6.43 Gb of raw reads with 18,967,988 paired-end reads were obtained from high-throughput sequencing. The raw data was filtered using Trimmomatic v.0.32 with default settings (Bolger et al. 2014). Then paired-end reads of clean data were assembled into circular contigs using GetOrganelle.py (Jin et al. 2018) with *Gentiana crassicaulis* (No. NC_027442) as reference. Finally, the cpDNA was annotated by the Dual Organellar Genome Annotator (DOGMA; http://dogma.ccbb.utexas.edu/) (Wyman et al. 2004) and tRNAscan-SE (Lowe and Chan 2016) with manual adjustment using Geneious v. 7.1.3 (Kearse et al. 2012).

The circular genome map was generated with OGDRAW v.1.3.1 (Greiner et al. 2019). Then the annotated chloroplast genome was submitted to the GenBank under the accession number MN463101. The total length of the chloroplast genome was 151,569 bp, with 37.8% overall GC content. With typical quadripartite structure, a pair of IRs (inverted repeats) of 25,471 bp was separated by a small single copy (SSC) region of 16,921 bp and a large single copy (LSC) region of 81,177 bp. The cp genome contained 114 genes, including 78 protein coding genes, 30 tRNA genes, and 4 rRNA genes. Of these, 18 genes were duplicated in the inverted repeat regions, 7 genes, and 6 tRNA genes contain one intron, while three genes (*ycf3*, *rps12* and *clpP*) have two introns.

To investigate its taxonomic status, a total of 20 cp genome sequences of Gentianaceae species were downloaded from the NCBI database used for phylogenetic analysis. After using MAFFT V.7.149 for aligning (Katoh and Standley 2013), a neighbor-joining (NJ) tree was constructed in MEGA v.7.0.26 (Kumar et al. 2016) with 1000 bootstrap replicates and five Oleaceae species (*Osmanthus yunnanensis*: MH745226, *Osmanthus delavayi*: MH745225, *Fraxinus mandshurica*: NC_041463, *Fraxinus excelsior*: NC_037446 and *Forsythia suspensa*: NC_036367) were used as outgroups. The results showed that *G. delavayi* was closely related to *G. stipitata* (Figure 1). Meanwhile, the phylogenetic relationship in Gentianaceae was consistent with previous studies and this will be useful data for developing markers for further studies.

**Figure 1.**
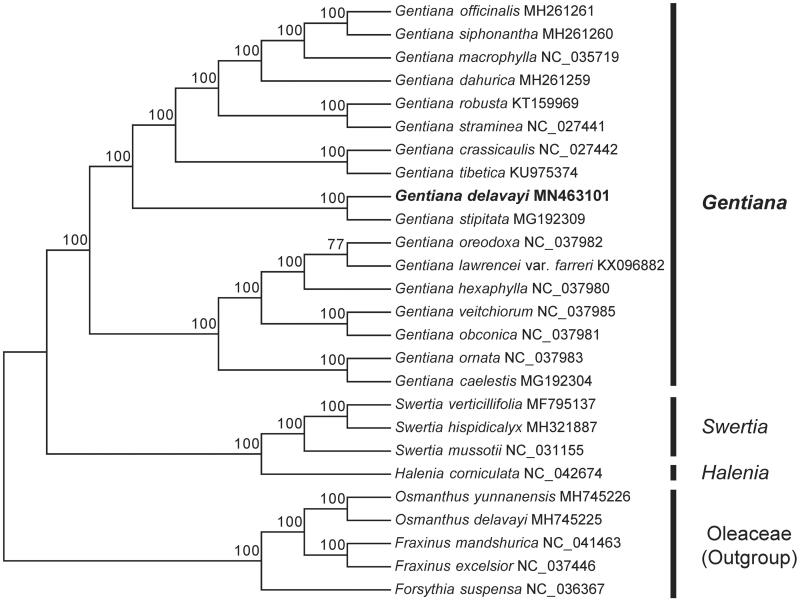
Neighbor-joining (NJ) tree of 21 species within the family Gentianaceae based on the plastomes using five Oleaceae species as outgroups.
